# NS3 protease from flavivirus as a target for designing antiviral inhibitors against dengue virus

**DOI:** 10.1590/S1415-47572010000200002

**Published:** 2010-06-01

**Authors:** Satheesh Natarajan

**Affiliations:** Department of Biochemistry, Faculty of Medicine, University of Malaya, Kuala LumpurMalayasia

**Keywords:** antiviral inhibitor, drug discovery, multifunctional protein, NS3, protease

## Abstract

The development of novel therapeutic agents is essential for combating the increasing number of cases of dengue fever in endemic countries and among a large number of travelers from non-endemic countries. The dengue virus has three structural proteins and seven non-structural (NS) proteins. NS3 is a multifunctional protein with an N-terminal protease domain (NS3pro) that is responsible for proteolytic processing of the viral polyprotein, and a C-terminal region that contains an RNA triphosphatase, RNA helicase and RNA-stimulated NTPase domain that are essential for RNA replication. The serine protease domain of NS3 plays a central role in the replicative cycle of dengue virus. This review discusses the recent structural and biological studies on the NS2B-NS3 protease-helicase and considers the prospects for the development of small molecules as antiviral drugs to target this fascinating, multifunctional protein.

## Introduction

The genus *Flavivirus* in the family *Flaviviridae* contains a large number of viral pathogens that cause severe morbidity and mortality in humans and animals (Bancroft, 1996). In endemic areas, dengue virus is transmitted by the main mosquito vector, *Stegomyia aegypti* (formerly *Aedes*), and circulates as a complex of four serotypes, DENV1 to DENV4 (Bancroft, 1996), that are all causative agents of dengue fever, dengue hemorrhagic fever and dengue shock syndrome. The high burden on public health care and the limited options of therapy for dengue diseases have stimulated efforts to identify and characterize potential viral drug targets, such as the non-structural protein 3 (NS3) serine proteases, and to design and evaluate antiviral inhibitors that are equally effective against the four dengue virus serotypes and related members of the *Flaviviridae* (Lindenbach and Rice, 2007).

The positive-sense flavivirus RNA genome of 11 kb forms a single open reading frame that is translated into an ~370 kDa polyprotein precursor containing the structural proteins C, prM, and E and seven non-structural proteins known as NS1, NS2A, NS2B, NS3, NS4A, NS4B and NS5. The polyprotein is co- and post-translationally cleaved by host cell proteases and the viral protease NS3 into three structural proteins (Capsid, premembrane and envelope proteins) that are components of the virion, and at least seven non-structural proteins involved in viral replication and maturation (Henchal and Putnak, 1990; Kautner *et al.*, 1996). The virus-encoded protease complex NS2B-NS3 is responsible for cleaving the NS2A/NS2B, NS2B/NS3, NS3/NS4A and NS4B/NS5 junctions and the Capsid protein.

Dengue diseases are caused by the four antigenically distinct dengue virus serotypes, DENV1-4. Current evidence suggests that the two virus-encoded non-structural proteins NS3 and NS5 that contain the key enzymes required for RNA replication are sufficiently conserved within the four serotypes to permit the design of compounds that will be active against all dengue virus strains and other related flaviviruses such as YFV, 70 WNV and JEV (Li *et al.*, 2005; Keller *et al.*, 2006). Since dengue virus and hepatitis C virus (HCV) are members of the *Flaviviridae*, it is interesting to compare the conditions that they cause: dengue produces an acute self-limiting illness whereas HCV provokes a chronic disease that can potentially lead to hepatocarcinoma. Given the short period of viremia, the question often posed is what benefit an antiviral compound would have in treating patients with dengue. Epidemiological studies have indicated that serious dengue diseases are often associated with high levels of circulating virus (Gubler *et al.*, 1981; Libraty *et al.*, 2002). Consequently, if dengue fever is diagnosed at an early stage through identification of the viral genome by RT-PCR or the presence of circulating NS1 protein in the blood, then antiviral treatment could be useful in limiting the viral load, thereby reducing the severity of the disease (Keller *et al.*, 2006). In the case of epidemics, one could also envision the use of prophylactic treatment to target groups of patients identified through decision tree algorithms based on simple clinical and hematological parameters (Tanner *et al.*, 2008).

**Figure 1:**
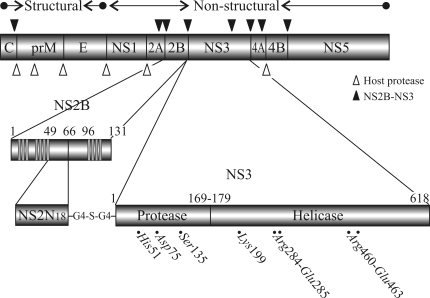
Schematic representation of the flaviviviral polyprotein with the cleavage sites processed by NS2B-NS3pro indicated by arrows. Also shown is the partition of the various functional domains along the primary sequence of NS2B-NS3. The regions of the NS2B proteins expected to associate with membranes are indicated as filled boxes. Evolutionarily conserved residues essential for NS3 enzymatic activities are indicated.

The identification of novel antiviral compounds active against dengue enzymes that are essential for viral replication required detailed 3D structural studies of the two multifunctional proteins NS3 and NS5. The recent determination of the 3D structures of the major replicative enzymes from flaviviruses, particularly the protein NS3, is discussed below. This protein contains a serine protease domain in the N-terminal region that requires the formation of a non-covalent complex with the membrane-bound cofactor NS2B for activity, and has ATP-driven helicase and RNA triphosphatase activities in the C-terminal region (Figure 1). Interestingly, NS3 is apparently also involved in virus assembly via mechanisms that are independent of the enzymatic functions outlined above (Khromykh *et al.*, 2001; Patkar and Kuhn, 2008).

## Dengue Virus Life Cycle

The dengue viral particle consists of a lipid bilayer surrounding a capsid that contains the 10.7 kb positive-strand RNA genome (Lindenbac *et al.*, 2007). The viral RNA contains a type I methyl-guanosine cap structure at its 5'end but is devoid of a poly-adenylate tail (Ray *et al.*, 2006). The first step in viral infection is the binding to a cell-surface receptor (Chu *et al.*, 2005; Fink *et al.*, 2006) that results in major structural changes in the surface envelope glycoprotein E, a class I viral fusion glycoprotein. Viral entry subsequently occurs via endocytosis, with acidification of the endosome (Roussel *et al.*, 2006) leading to the merging of host and virus lipid membranes (Bressanelli *et al.*, 2004; Modis *et al.*, 2004) and the release of viral genomic RNA into the host cell cytoplasm. The dengue virus genome encodes an uninterrupted open reading frame of 10,173 nucleotides that is translated into a polyprotein precursor 3391 amino acids long. The order of proteins in this precursor is: C-prM-E-NS1-NS2-NS2A-NS2B-NS3-NS4A-NS4B-NS5. The polyprotein is co- and post-translationally cleaved by host cell proteases and the viral protease NS3 into three structural proteins (Capsid, premembrane and envelope proteins) that are components of the virion and at least seven non-structural proteins involved in virus replication and maturation (Bera *et al;*, 2007). Once the viral polymerase NS5 has been synthesized and released from the polyprotein precursor, the viral RNA is transcribed, starting from the 3'end, into the complementary minus strand RNA that is then transcribed back into a positive strand. The transient dsRNA intermediate formed during this process must be separated into its individual strands in order to provide access to the NS5 polymerase for further rounds of viral genome replication (Bera *et al;*, 2007).

You *et al.* (2001) and Yon *et al.* (2005) provided detailed descriptions of the structural requirements for the RNA molecule that ensure viral RNA synthesis. These include a promoter region located as the 5' end of the genome to which the NS5 polymerase directly binds, as well as long range RNA-RNA interactions between stem-loop forming motifs located at the 5' and 3' ends of the viral genome that lead to its cyclization. NS3, which physically associates with the NS5 polymerase (Johansson *et al.*, 2001; You *et al.*, 2001), has an essential role in the viral life cycle since mutagenesis studies have shown that impairment of either its proteolytic or NTPase/helicase activity leads to a defective genome unable to produce infectious viral particles (Matusan *et al.*, 2001).

The most conserved flavivirus protein is NS5, which has a methyltransferase motif in the N-terminal domain and an RNA-dependent RNA polymerase in its C-terminal domain (Tan *et al.*, 1996). After processing of the viral proteins, most of the NS proteins associate with the 3'-UTR of viral RNA to form a replication complex for RNA synthesis. The association of protein C with genomic RNA on the cytosolic face of the endoplasmic reticulum membrane is the initial step of virion assembly. The particles are transported via the secretary pathway to the cell surface for release.

## The Ns2b-Ns3pro Complex

The serine protease domain of NS3 is located in the first 180 amino acids of the N-terminal region of this multifunctional protein (Figure 1). The NS3 protease, which is also considered a member of the flavivirin enzyme family (EC 3.4.21.91 and S07.001 Peptidase MEROPS peptidase database), has a tendency to form aggregates and is enzymatically inactive. The presence of a small activating cofactor protein is a prerequisite for optimal catalytic activity of flaviviral proteases with natural polyprotein substrates (Li *et al.*, 2005). This cofactor, which is a central hydrophilic portion (spanning residues 49-95) of the integral membrane protein NS2B, is required for viral protease activation (Falgout *et al.*, 1991; Keller *et al.*, 2006), including that of NS3 (Erbel *et al.*, 2006) (Figure 1). The NS2B - NS3 conjugate cleaves the precursor polyprotein at NS2A/NS2B, NS2B/NS3, NS3/NS4A and NS4B/NS5 junctions, as well as at internal sites within C, NS2A, NS3 and NS4A (Falgout *et al.*, 1991). NS2B consists of seven domains (I - VII) that can be separated on the basis of their relative hydrophobicity (Falgout *et al.*, 1991). The hydrophobic core residues belonging to domain IV (G69-E80) were proposed to interact with NS3pro. This domain is flanked by two hydrophilic stretches (domains III and V). Studies using mutant plasmids transfected into cells have shown that a fragment of 40 residues encompassing domains III to V is the minimal region necessary for inducing the protease activity of NS3pro (Speroni *et al.*, 2007). In addition, co-immunoprecipitation experiments have shown that the NS2B - NS3 association is also mediated by this hydrophilic region (Chambers *et al.*, 1991).

Comparison of the kinetic properties of NS3 and NS2B - NS3 suggests that NS2B generates additional specific interactions with the substrate residues P2 and P3 (Chambers *et al.*, 1990). The molecular mechanism by which the NS2B cofactor stimulates protease activity is unknown, although comparison with an analogous HCV protease has provided some structural and mechanistic explanations for activation of the dengue virus protease by its cofactor. However, unlike the HCV NS3 protein, the cofactor activity for DEN-2 NS3pro cannot be supplied in trans with a small peptide derived from NS2B (Bartenschlager *et al.*, 1995). Other serine proteases (subtilisin, α-lytic protease) also require a pro-region, such as NS2B, to mediate folding that leads to the active form. In these cases, once the protein is folded, the necessary pro-region does not remain bound to the active enzyme.

The active protease has been successfully expressed by fusing the 5' end of the NS2B viral cofactor spanning residues 49-95 (CF40) of DENV2 to amino-acids 1-169 of the NS3 protein via a flexible glycine linker (Gly4-Ser-Gly4) linker (Leung *et al.*, 2001). Leung *et al.* (2001) cloned and expressed the protease (CF40-Gly4-Ser-Gly4-NS3pro) from all four dengue serotypes (DENV 1-4) and used the *in vitro* assay described by Erbel *et al.* (2006) to screen a tetrapeptide library containing more than 130,000 substrates. The tetrapetide benzoyl-norleucine (P4) - lysine (P3) - arginine (P2) - arginine (P1) - ACMC (Bz-Nle-Lys-Arg-Arg-ACMC) was identified as the optimal substrate with the steady state kinetics parameter 176 *k*cat/*K*m of 51,800 M^- 1^ s^- 1^, which is > 150-fold more sensitive than for other peptides. This sensitivity allowed the assay to be scaled down for high-throughput screening (Yusof *et al.*, 2000). The tetrapeptide aldehyde Bz-Nle-Lys-Arg-Arg-H, a competitive inhibitor of the protease domain and of the full length NS3protein with its cofactor (CF40-Gly4-Ser-Gly4-NS3FL) (*K*i of 5.8 μM and 7.0 μM, respectively) has been used in structure - activity relationship (SAR) experiments to identify more potent inhibitors. This approach has allowed the development of compounds based on strategies used to overcome the shallow binding pocket of the HCV protease. A high resolution analysis of the interaction between NS3 and NS2B using the tetrapeptide aldehyde inhibitor indicated that the central hydrophilic portion of the NS2B cofactor refolds to form an important component of the protease catalytic site (Erbel *et al.*, 2006). This analysis provides a basis for screening drugs that can target flaviviral proteases, *e.g.*, the apo-enzyme from DENV2 and a complex formed by the WNV NS2B-NS3Pro enzyme and Bz-Nle-Lys-Arg-Arg.

**Figure 2:**
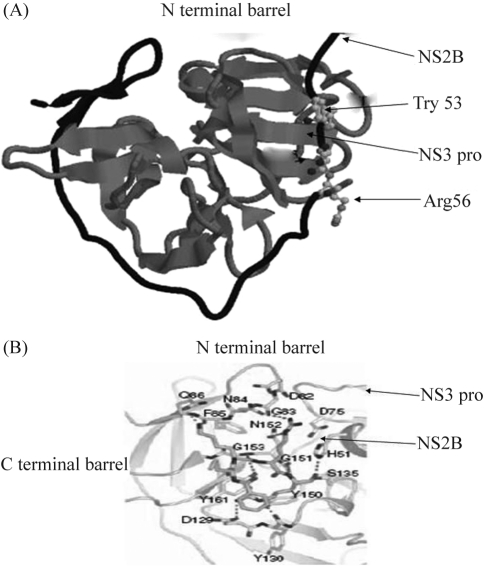
(A) Schematic representation of the NS2B-NS3 protease showing the apo-enzyme from DENV2 with the NS2B cofactor in dark shade. The WNV residues Trp_53_-Arg_56_ are represented by sticks. (B) NS2B Pro and NS2B showing important residues.

In both structures, the N-terminal part of the NS2B cofactor (residues 49-66) forms a β-strand that is inserted into the N-terminal β-barrel of the protease, thereby concealing hydrophobic residues from the solvent and stabilizing this domain; WNV residues Trp_53_-Leu_54_-Glu_55_-Arg_56_-Ala_57_-Ala_58_ form a β-strand to the N-terminal β-barrel of the NS3 protease (Figure 2). This explains the observed strong tendency for NS3pro and FL NS3 to aggregate when the strand contributed by NS2B is absent in synthetic constructs. In this respect, the N-terminal of NS2B has a chaperone-like role in stabilizing NS3. In contrast, the conformation of the C-terminal part of the NS2B cofactor is totally different in the apo- and inhibitor-bound enzymes: a large rearrangement brings residues 66-88 of NS2B in close proximity to the substrate-like inhibitor to form a belt that braces the second chymotrypsin-like β-barrel. Amino-acids Arg78-Leu87 of the NS2B cofactor affect the formation of the active site by contributing a β-hairpin that inserts into the C-terminal β-barrel thereby reorienting residues for substrate recognition. Nuclear magnetic resonance experiments have shown that this C-terminal domain is disordered in the absence of the substrate-like inhibitor but becomes ordered upon binding (Erbel *et al.*, 2006). Thus, the conformation adopted by the C-terminal segment of NS2B in the apo-enzyme crystal structure is probably only one amongst those that are accessible in solution and appears to be dictated by crystal packing forces. The dual role of the 40 amino acid NS2B cofactor region in stabilizing the protein and forming the active site of the protease is indeed unique to flaviviruses and distinguishes it from HCV NS3 protease that requires only a 12 amino acid sequence from NS4A to form the active protein.

The ability of aprotinin to occupy all of the specificity pockets of the protease with the subsequent formation of a fully formed oxyanion hole has provided a complete view of the enzyme-substrate Michaelis complex for a flavivirus protease (Speroni *et al.*, 2007). Analysis of the resulting structures has provided new opportunities for discovering flavivirus-specific drugs that could interfere with protein - protein interactions required for activation of the protease, in addition to the development of competitive inhibitors. Aleshin *et al.* (2007) reported a search for small molecular weight inhibitors of flaviviral NS3 protease. Based on initial screening experiments, Johansson *et al.* (2001) selected a common scaffold that was further improved by targeted chemical modification during lead optimization to identify a class of [5-amino-1-(phenyl) sulfonyl-pyrazol-3-yl)] compounds that may function by blocking the NS2B binding pocket within NS3, thereby preventing the interaction between the two proteins that is needed for protease activity. Structural studies of the NS2B-NS3 complex from WNV and DENV, together with assays using specific substrates, provide a useful approach for identifying and developing novel, selective NS3 protease inhibitors.

## The Ns3 Ntpase/Helicase

The 3' end of NS3 forms the RNA helicase domain, an ATP-driven molecular motor that modifies the topology of nucleic acids. The specific role of the helicase domain in the viral life cycle is unclear, although the enzyme is generally believed to separate transient, intermediate dsRNA formed during polymerization catalyzed by NS5 viral polymerase into its individual strands (Malet *et al.*, 2007); this strand separation facilitates further rounds of replication and transcription. The helicase domain may also facilitate viral RNA replication by disrupting secondary structures formed by the ssRNA template or by displacing other host or viral proteins bound to dsRNA. The ATPase activity of NS3 is stimulated by RNA, thus highlighting the extensive cross-talk between the various catalytic centers of the NS3 enzyme. Wu *et al.* (2005) studied the NS3 helicase/nucleoside triphosphatase catalytic domains of DENV2 and YFV. The proteins consist of three subdomains of approximately 140 residues. All seven of the 283 amino-acid sequence motifs, including motif I (also known as Walker A, the phosphate binding loop or P-loop), that have been identified in members of the SF2 superfamily of helicases (Gorbalenya and Koonin, 1993) are located in subdomains 1 and 2. Despite the very low level of sequence identity between them, both subdomains adopt the same μ/β fold initially identified in the RecA protein (Xu *et al.*, 2005). The ATP binding site is located between the two subdomains. The mode of ATP binding was initially visualized through the suking of ATP (even though only ADP was located in the binding site, as shown by analysis at very high crystallographic temperatures) and by comparison with models of other ATP-driven DNA helicases. These analyses suggested that ATP was bound primarily through its triphosphate moiety via contacts mediated by residues emanating from the P-loop and DEAH motif II (or Walker B motif). In DENV 4 helicase bound to ADP and AMP PNP, adenylyl imidodiphosphate contact with the 3'OH group of ribose is direct whereas contact with the 2'OH is mediated by a water molecule. The ATP-binding domain is structurally well conserved when compared to other SF2 helicases, including that of HCV, with subdomian 3 of the flavivirus helicase being the most divergent (Xu *et al.*, 2005). Kapoor *et al.* (1995) attempted to reconcile the structural diversity among the respective polymerases and the way they associate with NS3 subdomain 3 to form the replication complex. Kim *et al.* (1998) proposed that a groove located at the interface between the three subdomains housed a single-stranded nucleic acid substrate of approximately 6-8 ribonucleotides that would form the 3' overhang needed by the enzyme to translocate along the substrate during unwinding. Sampath *et al.* (2001) used site-directed mutagenesis of full-length NS3 to examine the effect of Ala substitutions in subdomain 2 on the ATPase, helicase and RTPase activities of this protein. Since an excellent correlation was found between the ATPase and RTPase activities for the various mutants studied the two reactions were proposed to share the same site, in agreement with previous studies (Benarroch, 2004). A less encouraging finding for drug design was the existence of residual helicase activity that was ATPase-independent (Bartelma *et al.*, 2002). The limited number of interactions provided by the sugar and base moieties in the ATP binding site suggests that the ATP binding site may not be a good target for the design of competitive inhibitors. In addition, there are important questions that still need to be addressed, such as the number of base pairs separated at each step and the amount of ATP needed for the reaction. Future investigations should aim to precisely define the various structural states assumed by the NS3 enzyme during catalysis. This information could be particularly useful in the design of “allosteric inhibitors” aimed at preventing the structural transitions that occur during catalysis.

## Conclusions

Although there are still no specific vaccines or chemotherapeutic regimes for the prevention and treatment of dengue fever and dengue hemorrhagic fever, in recent years there has been substantial progress in our understanding of the life cycle of dengue virus, the various stages of which represent potential targets for the development of novel antiviral drugs. NS3 protein is a particularly interesting molecular target for antiviral compounds because of it central role in the viral life cycle. The structural similarities between the active protease and helicase of NS3 suggest that a compound that could hamper the dynamics of this multifunctional enzyme would be attractive. Such a compound could for instance target the entrance of the RNA binding tunnel (towards the 5' end of the RNA substrate) which is lined by two mobile helices, or prevent protein - protein interactions between NS3 and NS2B. A challenging aspect in the search for potent, selective antiviral drugs that interfere with multifunctional NS3 is the design of appropriate assays for druggable sites that are relevant for viral replication *in vivo*. Inhibitors of NS3pro should also be of great benefit in combating infections by other Flaviviruses, as well as Japanese encephalitis virus and West Nile virus. The development of such drugs requires a more informed structure-based drug discovery program. A further consideration includes the cost of drug synthesis which should not make the price of the final product prohibitive to poor patients in developing countries.
